# Methanolic Extracts from Cultivated Mushrooms Affect the Production of Fumonisins B and Fusaric Acid by *Fusarium verticillioides*

**DOI:** 10.3390/toxins12060366

**Published:** 2020-06-02

**Authors:** Daniel Merel, Jean-Michel Savoie, Gerardo Mata, Dulce Salmones, Carlos Ortega, Vessela Atanasova, Sylvain Chéreau, Juan L. Monribot-Villanueva, José A. Guerrero-Analco

**Affiliations:** 1 Red Manejo Biotecnológico de Recursos (RMBR), Instituto de Ecología (A.C), Xalapa 91073, Mexico; danielmerel@hotmail.com (D.M.); dulce.salmones@inecol.mx (D.S.); carlos.ortega@inecol.mx (C.O.); 2 Red Estudios Moleculares Avanzados (REMAV), Instituto de Ecología (A.C), Xalapa 91073, Mexico; juan.monribot@inecol.mx; 3 INRAE, Mycology and Food Safety (MycSA), F-22882 Villenave d’Ornon, France; vessela.atanasova@inrae.fr (V.A.); sylvain.chereau@inrae.fr (S.C.)

**Keywords:** mycotoxins, biocontrol, mushroom metabolomics, phenols, basidiomycetes

## Abstract

The maize pathogen *Fusarium verticillioides* and their mycotoxins cause damage to plants, animals, and human health. This work aimed to evaluate the effect of crude extracts (CEs) from *Agaricus subrufescens*, *Lentinula edodes*, and *Pleurotus ostreatus* fruiting bodies on in vitro production of biomass and mycotoxins by two strains of *F. verticillioides*. Stipes and pilei were separated before extraction for *A. subrufescens* and *L. edodes*. Comparative metabolomics and dereplication of phenolic compounds were used to analyze all CEs. Mushroom CEs did not significantly inhibit the production of mycelial biomass at concentrations of 2 mg mL^−1^. CEs from *A. subrufescens* (stipes and pilei) and *L. edodes* pilei inhibited the production of fumonisins B1 + B2 + B3 by 54% to 80%, whereas CE from *P. ostreatus* had no effect. In contrast, CE from *L. edodes* stipes dramatically increased the concentration of fumonisins in culture media. Fusaric acid concentration was decreased in cultures by all CEs except *L. edodes* stipes. Differences in phenolic composition of the extracts may explain the different effects of the CE treatments on the production of mycotoxins. The opposing activities of stipes and pilei from *L. edodes* offer an opportunity to search for active compounds to control the mycotoxin production by *F. verticillioides*.

## 1. Introduction

Both governmental and non-governmental organizations responsible for food safety of developed and developing countries are concerned for the contaminations of maize (*Zea mays* L.) by fungi and their mycotoxins. This contamination is the source of a variety of negative consequences to the health of livestock and humans feeding on maize [1,2]. *Fusarium verticillioides* (Sacc.) Nirenberg is commonly isolated from diseased maize plants with symptoms of ear and kernel rot from tropical areas [3]. This species is known to be a producer of fumonisins and fusaric acid (FA) mycotoxins that are found in maize kernels [4]. More than fifty fumonisins have been reported and clustered into A, B, C, and P groups based upon their chemical structures and phytotoxicity level, with fumonisins B1 (FB1), B2 (FB2), and B3 (FB3) as the most common types found. Specifically, FB1 can comprise from 70% to 80% of the total fumonisins [5,6] found when the fungus is cultured on maize or in liquid medium. Alarmingly, epidemiological studies have associated a higher frequency of human esophageal cancer with the presence of FB1 within foods in China, Iran, and South Africa [7,8]. According to the International Agency for Research on Cancer, FB1 is classified as a possible carcinogenic to humans (group 2B) [9]. Furthermore, maternal consumption of fumonisins in maize grains or processed food made from maize during early gestation cause spinal bifida and anencephalic births [10,11]. Additionally, the fumonisin intake through contaminated feed is responsible for animal fatal toxicosis, such as leukoencephalomalacia in horses [12,13] and pulmonary edema in swine [14]. FA, the other mycotoxin produced by *F. verticillioides* and commonly detected in grains, is known for its high phytotoxicity. It can inhibit maize seedling development [15] and exhibit adverse effects on tissues of cotton, banana, and tomato plants [16,17,18]. Recent studies have found that FA induces DNA damage and necrosis in human hepatocellular carcinoma cells [19,20], apoptosis in human cancerous esophageal cells [21], and mitochondrial stress in human cervix carcinoma cells [22]. FA also shows detrimental outcomes towards animals by causing negative neurochemical effects in rats [23] and teratogenic effects in zebrafish [24], and it has been reported to synergize the toxicity of FB1 [25].

Fungal growth and mycotoxin accumulation in grains is affected by climatic conditions during pre-harvest and harvest stages. Furthermore, inadequate conditions of grain storage and transport can promote the toxigenic fungi growth and contamination [26]. A current strategy proposed to control phytopathogenic fungi and their mycotoxin production is the application of natural products that inhibit the growth of the toxic fungi and/or the production of pernicious metabolites. This approach is suitable for applications in agriculture due to their low toxicity that minimizes the potential for environmental impacts to agriculture and harmful effects to human health [27].

Phenolic compounds are specialized metabolites that have been effective to inhibit the mycelial growth and toxin production of fungi [28,29] and generally are known to have low toxicity. Namely, addition of phenolic acids [30,31] and flavonoids [32] in culture media reduced fumonisin production of *F. verticillioides*. Basidiomycete mushrooms also produce antifungal compounds [33]. In a review of the literature from 1999 to 2012, extracts from 52 species had been reported for their antifungal properties [34]. More than 80% of these species are edible mushrooms, since their extracts might be generally recognized as safe for consumption. Most of the studies have focused on the inhibition of fungal growth by crude or partially simplified extracts and have not identified specific compounds responsible for their antifungal activity. Only a few of these were conducted to identify the effects of the extracts from these edible mushrooms against mycotoxin production, leaving these organisms untapped sources for preventing and reducing mycotoxins in cereal products [35]. *Agaricus subrufescens* Peck, *Lentinula edodes* (Berk.) Pegler, and *Pleurotus ostreatus* (Jacq.) P. Kumm. (commonly known as almond mushroom, shiitake, and oyster mushroom, respectively) are gourmet mushrooms that are cultivated worldwide. The aim of this work was to evaluate the potential of crude extracts (CEs) obtained from fruiting bodies of *A. subrufescens*, *L. edodes*, and *P. ostreatus* to inhibit the production of fumonisins and FA by *F. verticillioides*, and to begin to identify specific chemical compounds responsible for this bioactivity.

## 2. Results

### 2.1. Extraction Yields

The yield of methanolic extracts varied with samples, from 13.2% in stipe of *A. subrufescens* to 29.6% in whole sporophore of *P. ostreatus* (Table 1). In *L. edodes* pileus, an amorphous powder spontaneously precipitated during the evaporation of methanol. The precipitate (14.3% of the initial biomass) was separated from its liquors by decantation before drying for obtaining CE used for biological tests and metabolomics.

### 2.2. Effect of CEs on Fungal Growth of F. verticillioides Fv63 and MY3 Strains

**Figure 1:**
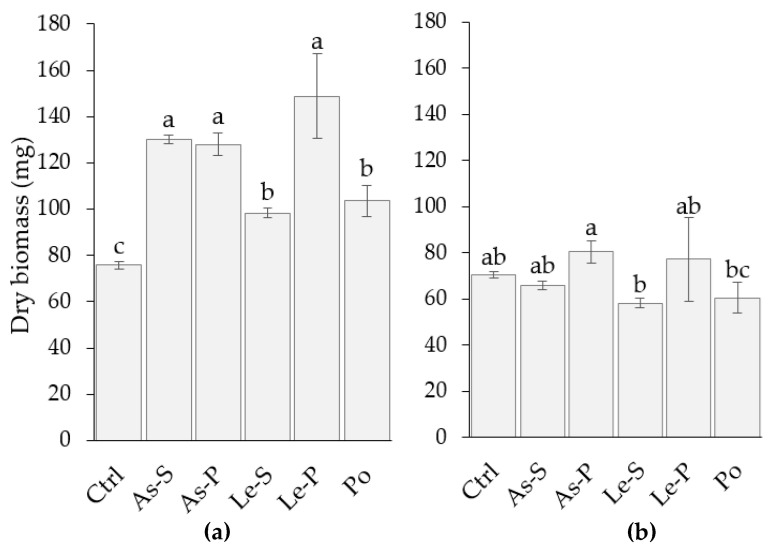
Fungal biomass of *F. verticillioides* strains produced in liquid media supplemented with 2 mg mL^−1^ crude extracts. (**a**) Represents fungal biomass measurements for all treatments of strain Fv63. (**b**) Represents fungal biomass measurements for all treatments of strain MY3. Fungal biomass data was recorded for three replicates and the ± standard deviation is represented by the error bars. Letters (a, b, and c) indicate significant differences between treatments and control, according to Tukey’s Test (α = 0.05). Extracts are represented by the following abbreviations: Control (Ctrl), CEs from *A. subrufescens* stipe (As-S) and pileus (As-P), *L. edodes* stipe (Le-S) and pileus (Le-P), *P. ostreatus* sporophore (Po).

All the quantities of mycelial biomass produced by *F. verticillioides* strain Fv63 were significantly higher in cultures treated with CEs of *A. subrufescens* stripe (As-S), *A. subrufescens* pileus (As-P), *L. edodes* stipe (Le-S), *L. edodes* pileus (Le-P), and *P. ostreatus* sporophore (Po) when compared to the control (Figure 1a). On the other hand, treatment of the MY3 strain of *F. verticillioides* with these CEs demonstrated no effect on growth compared to the control (Figure 1b).

### 2.3. Effect of CEs on Fumonisin Production by F. verticillioides Fv63

**Figure 2:**
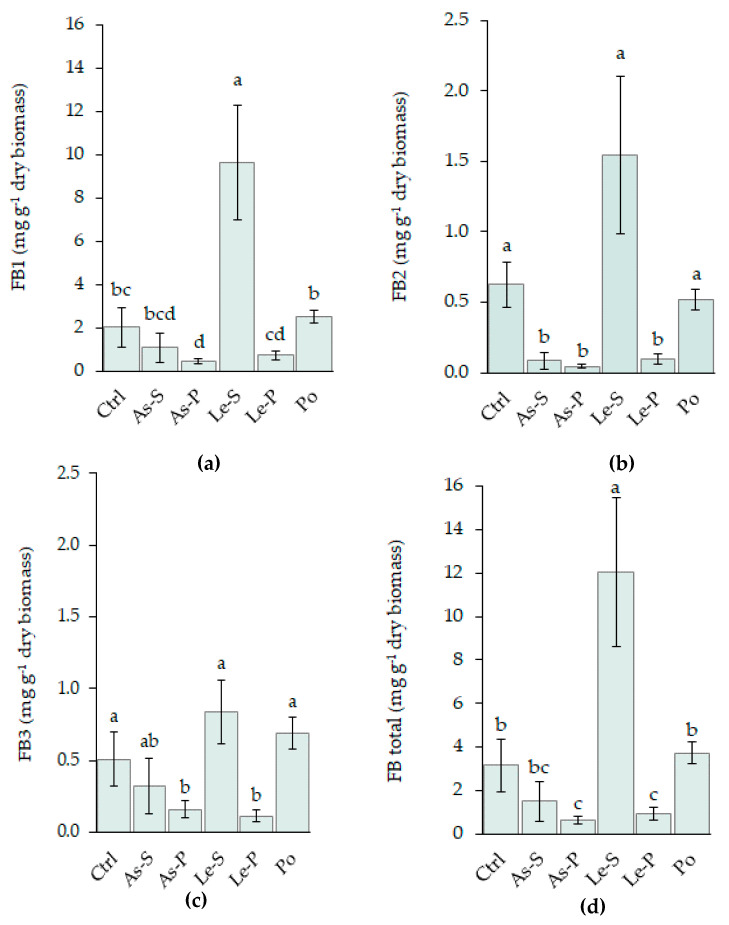
Effects of CE treatments of As-S, As-P, Le-S, Le-P, and Po on fumonisins B1 (**a**), B2 (**b**), B3 (**c**), and total (B1 + B2 + B3) fumonisin (**d**) produced by *F. verticillioides* (Fv63 strain) in glucose, amylopectin, yeast extract, and peptone (GAYEP) medium supplemented with 2 mg mL^−1^ CEs. Data are represented as the mean mycotoxin production of three replicated cultures and three measurements per cultures with the ± standard deviation represented by error bars. Letters (a, b, and c) indicate significant differences between treatments and control according to Tukey’s Test (α = 0.05).

CE from As-P was demonstrated to inhibit the FB1 and FB2 production of the Fv63 strain by 79% and 92% respectively, and demonstrated similar activity against FB3 production (69%) (Figure 2). As-S significantly inhibited FB2 production (86%) and decreased FB1 (46%) and FB3 (36.8%) concentrations. CE from Le-P inhibited the production of the three fumonisins (64.5% for FB1, 84.37% for FB2, and 77.6% for FB3), while CE from Le-S significantly increased their production (Figure 2a–c). FB1 + FB2 + FB3 production was inhibited by As-P and Le-P treatments (80.2% and 70.6%, respectively), whereas Le-S increased their accumulation by 4-fold (Figure 2d).

**Figure 3:**
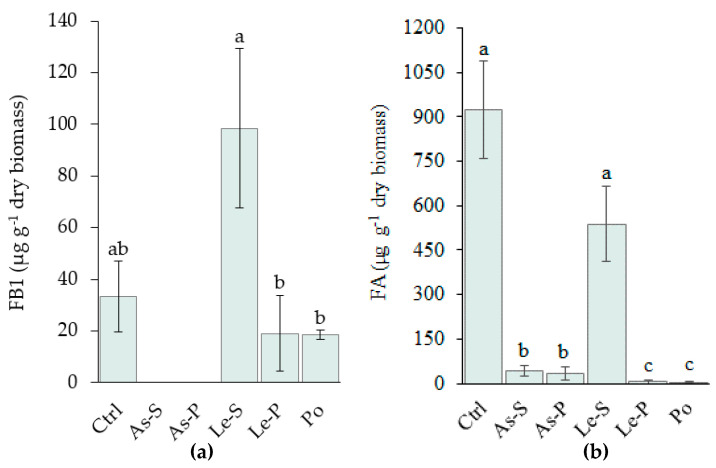
FB1 and FA produced by *F. verticillioides* (MY3 strain) in glucose, amylopectin, yeast extract, and peptone (GAYEP) medium supplemented with 2 mg mL^−1^ CEs. (**a**) FB1 production versus treatment with different CEs, (**b**) FA production versus treatment with different CEs. Data are represented by the mean of three replicated cultures and three measurements of FB1 or FA production and error bars represent the ± standard deviation. Letters (a, b, and c) indicate significant differences between treatments and the control, according to Tukey’s Test (α = 0.05). Extracts were: Control (Ctrl), CEs from *A. subrufescens* stipe (As-S) and pileus (As-P), *L. edodes* stipe (Le-S) and pileus (Le-P), *P. ostreatus* sporophore (Po).

Under these conditions, the MY3 strain produced less fumonisins than Fv63 and detection limits only allowed the quantitation of FB1 in this strain. Treatment with As-S and AS-P extracts reduced the FB1 production below the limit of detection, while no difference in FB1 production was demonstrated by treatment with the other CEs compared to the control. Le-S treatment was found to increase the concentration of FB1 by 200%, whereas Le-P decreased it by 43%. The difference between Le-S and Le-P was found to be significant (Figure 3a).

### 2.4. Effect of CEs on Fusaric Acidproduction by F. verticillioides MY3

FA production by MY3 was almost completely inhibited by treatment with the CEs in the following order Po = Le-P > As-P = As-S (99.5–95.3%). FA production was not significantly affected by treatment with *L. edodes* stipe CE (Figure 3b).

### 2.5. Metabolomic Analysis and Chemical Profiling of the CEs

**Figure 4:**
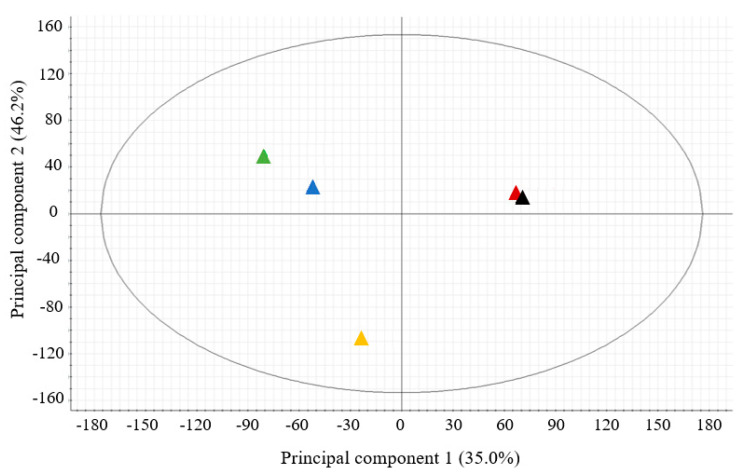
Principal component analysis (PCA) of ions detected in the CEs from *A. subrufescens* (▲pileus and ▲stipe), *L. edodes* (▲pileus and ▲stipe), and *P. ostreatus* (▲whole sporophore).

The metabolome similarities of all CEs were evaluated by a principal components analysis (PCA) and a fold change analysis that consider each feature (retention time/mass-to-charge ratio (*m*/*z*) pairs). The sample grouping as the result of PCA showed significant differences in chemical composition of the CEs of the three species of mushroom within the first two principal components (PC1 and PC2), which explains 46.2% and 35% respectively, of the total variance (Figure 4). Interestingly, only weak intraspecific differences were observed in the PCA analysis of CEs from As-s and As-P, while in *L. edodes*, the compositions of the Le-S and Le-P were found to be different (Figure 4).

**Figure 5:**
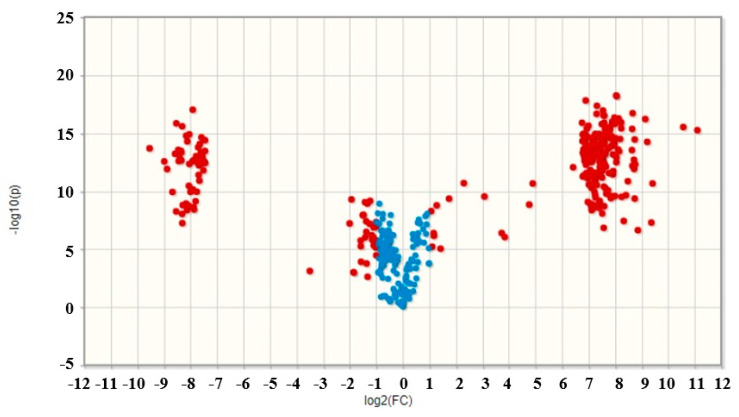
Volcano plot of ions detected in *L. edodes* CEs. Ions with significantly different abundance (fold change > 2) between the samples (●), ions with no significant difference in abundance between the samples (●).

Since biological activities and chemical compositions of CEs from Le-P and Le-S were found to be different, further investigations were performed on these extracts. 349 ions were detected in Le-P CE whereas 242 ions were detected in Le-S CE. Of these ions, 156 were detected at a similar abundance (Figure 5). Further metabolomic analysis allowed for the putative identification of ions that significantly differed between these samples. Adenine, L-phenylalanine, 3-amino-2-naphthoic acid and (22E,24x)-ergosta-4,6,8,22-tetraen-3-one were metabolites tentatively identified to be present only in Le-P, and farnesyl acetone was only identified in Le-S (Table 2).

### 2.6. Dereplication of Phenolic Compounds

The analysis detected five phenolic compounds in the CEs, one anthraquinone and four phenolic acids (Table 3). Chrysophanol was the most abundant compound contained in all sporophore structures (130.3–26,226.2 μg g^−1^) followed by protocatechuic acid (6.2–1245.9 μg g^−1^), shikimic acid (24.3–44.6 μg g^−1^), and 4-hydroxybenzoic acid (6.2–36.9 μg g^−1^). CE from Po, Le-S, and Le-P were the only samples that contained 4-hydroxyphenylacetic acid (5.6, 5.2, and 9.1 μg g^−1^, respectively). The lower concentrations of phenolic compounds detected were for sinapic acid in Po, Le-P, and As-S (5.0, 7.7, and 8.2 μg g^−1^, respectively). Finally, Po was the only CE that was found to contain *trans*-cinnamic acid at a low concentration (5.0 μg g^−1^) (Table 3).

## 3. Discussion

In the present work, we evaluated crude methanolic extracts of three edible mushroom species *Agaricus subrufescens*, *Lentinula edodes*, and *Pleurotus ostreatus* against the culture media from two strains of *Fusarium verticillioides*. Antifungal activity of mushroom extracts and isolated compounds have been shown for several species, which are mainly edible mushrooms, since they were the most studied because their derivatives might be generally recognized as safe for use [33]. Culture filtrates of *A. subrufescens* and *L. edodes* inhibited the mycelial growth of different phytopathogenic fungi such as *Fusarium solani* and *F. oxysporum* [37,38]. An extract from *A. subrufescens* fruiting bodies reduced growth of saprophytic fungi *Aspergillus* spp., *Penicillium* spp., and *Trichoderma* spp. [39]. Additionally, water extracts from spent mushroom substrates of *L. edodes* suppressed mycelial growth of the rice blast fungus *Pyricularia oryzae* [40]. In addition, ethanolic extracts of *Hydnum repandum*, a mycorrhizal species commonly known as the sweet tooth or hedgehog mushroom and broadly distributed in Europe and Asia, had strong antifungal effects on various *Fusarium* species, including *F. verticillioides* [41]. Nonetheless, to date, there are no previous studies that showed inhibition of *F. verticillioides* by extracts from *A. subrufescens*, *L. edodes*, or *P. ostreatus* [35]. The concentration of CEs used in this study was selected based on the previous report of Stojkovic et al. [39], who identified antifungal activity at a concentration around 2 mg mL^−1^ of a methanolic extract from *A. subrufescens* (or *A. brasiliensis*) fruiting bodies, and after a preliminary concentration-response analysis on both mycelial growth and mycotoxin production, using whole fruiting bodies of *A. subrufescens*. In our study, we did not observe any growth inhibition of the two *Fusarium* strains, and instead observed growth-stimulating effects of the cultured Fv63 treated with all the CEs. Extracts from mushrooms are considered to be rich in phenolic compounds and these molecules are thought to be the source of their anti-fungal and antioxidant activities [42]. It has been established that the effects of phenolic acids such as ferulic acid demonstrated dose-dependent effects on *F. verticillioides* growth, significantly reducing growth upon treating the culture with 20 mM of this compound and stimulating growth at 1.0 mM treatments [43]. The low concentrations of each phenolic compound, including ferulic acid in our treatments of the culture media, might be at the origin of the observed absence of inhibition on mycelial growth in the present work. Treatment of cultures with higher concentrations of these extracts may result in the inhibition of fungal biomass growth.

The main objective of this study was to search for inhibiting effects of CEs and their chemical composition on mycotoxin production independent of mycelial growth. Treatment of culture media of the Fv63 strain of *F. verticillioides* with 2 mg mL^−1^ CEs of pileus and stipe tissues of *A. subrufescens*, *L. edodes*, and *P. ostreatus* resulted in varied effects on FB1, FB2, FB3, and total fumonisin concentration depending on mushroom species and tissue. CEs of As-P and As-S generally inhibited fumonisin production at varying levels. CEs of *L. edodes* demonstrated dramatically different effects on fumonisin production depending on the tissue, with pileus extract demonstrating significantly inhibited production and stipe extract stimulating their production. *P. ostreatus* was found to have no significant effect on the fumonisin production. FB1 production in MY3 was inhibited by treatment with As-S and As-P extracts. Treatment with Le-S and Le-P extracts had opposing effects on FB1 production by the MY3 strain, with Le-S stimulating FB1 production and Le-P having no effect. The effects of the CEs on FA production were also evaluated in MY3. All extracts, except for Le-S, were found to significantly inhibit FA production. Given the varied effects of the extracts on fumonisin and FA production as the result of tissue and mushroom species, we hypothesized that chemical differences in phenolic composition and concentration were responsible in part for these differences in the biological activity.

To explore this hypothesis, we subjected the CEs to metabolomic analysis using liquid chromatography coupled to accurate mass spectrometry. A PCA of each CE revealed significant differences between the chemical composition of the CEs that corroborated the trends in biological activity that were observed. All three mushroom species extracts were found to have distinct metabolomic profiles, and variation between pileus and stipes of *L. edodes* were also distinctly different. Molecular annotation of the metabolomic profiles revealed differences in the phenolic composition between these extracts. Phenolic compounds are some of the specialized metabolites that have been identified as responsible for *F. verticillioides* growth inhibition [28,30,44]. These lipophilic compounds are thought to diffuse through the fungal membrane, penetrate the cell, and interfere in metabolic pathways involved in cell wall and membrane formation and transmembrane transport [45]. It has been reported that lipophilic compounds displayed a more efficient antifungal activity on other *Fusarium* species, such as *F. graminearum* [46]. The phenolics are also known to modulate the production of mycotoxins by *Fusarium* spp. [47], for instance fumonisins [28,48], but with variations among strains and species [30]. We identified and quantified the following phenolic acids in the CEs of *A. subrufescens*, *L. edodes*, and *P. ostreatus*: shikimic acid, *trans*-cinnamic acid, protocatechuic acid, 4-hydroxybenzoic acid, sinapic acid, 4-hydroxyphenylacetic acid, and the anthraquinone chrysophanol. According to Metabolomics Standards Initiative, these compounds were identified with a high-level confidence (level 1) based upon multiple dimensions of analysis [36]. 4-Hydroxybenzoic and protocatechuic acids have been previously reported in these three edible mushrooms [49,50,51] and were also identified in the current study. 4-Hydroxyphenylacetic acid were only identified in CEs from *P. ostreatus*, *L. edodes* stipe, and pileus. Koutrotsios et al. [52] also described that *P. ostreatus* contained 4-hydroxyphenylacetic acid; however, we report for the first time this compound in the extract of the *L. edodes* sporophore. Among the identified metabolites, chrysophanol was the most abundant phenolic compound contained in the three mushroom species. To date, this is the only report of this compound in the extracts of *A. subrufescens*, *L. edodes*, and *P. ostreatus*. Additionally, the presence of sinapic acid has not been reported before in CEs of *L. edodes* and *A. subrufescens*, but it has been previously described in extracts of *P. ostreatus* [53].

Other phenolic compounds present but not identified in the extracts might have contributed to their antimycotoxin activity. For instance, chlorophorin, iroko [28], vanillic acid [48], ferulic acid [54], chlorogenic, and caffeic acids [30] have been reported as FB1 inhibitors. As for the effects on mycotoxin production and mycelial growth, these effects were dose-dependent and perhaps, strain-dependent, in the present study. Ferrochio et al. [43] showed that ferulic acid activated the production of fumonisins at low concentrations (1 or 10 mM) while it inhibited them at higher levels (20–25 mM). In addition, no effect of caffeic acid was observed [43], contrary to the results of Atanasova-Pénichon et al. [47]. In CEs, interactions between phenolic compounds and other components might complexify the response of the fungi when compared to pure compounds.

The differences between the extracts from stipe and pileus of *L. edodes* in their effects on mycotoxins are noteworthy, especially for the production of FB1, FB2, FB3, and the sum of these compounds, as well as for the production of FA, whereas no significant difference was observed between extracts from stipe and pileus of *A. subrufescens*. Differences in activity between extracts from stipes and pilei of a same species are probably due both to differences in composition and concentrations of phenolic compounds and to the antioxidant activity of these extracts. In a previous study where a white hybrid and two wild strains of *Agaricus bisporus* were cultivated simultaneously under the same conditions, the gills had more than three-fold higher phenolic content than the other parts of the fruiting body [55]. As discussed above, a same phenolic acid might activate the production of fumonisins at low concentrations and inhibit it at higher ones [43,47]. The main differences between stipe and pileus of *L. edodes* observed here were for higher concentrations in sinapic acid, 4-hydroxy-benzoic acid, and 4-hydroxy-phenylacetic acid in CEs from pileus. Zabka and Pavela [56] documented the antifungal activity of sinapic acid against *F. verticillioides* and other mycotoxigenic fungi but did not search for effects on mycotoxins. Also, hydroxy-benzoic and 4-hydroxy-phenylacetic acids inhibited the growth of *F. oxysporum* and *Phytophthora capsici* respectively, among other phytopathogenic fungi [57,58]. At the concentrations of the CEs used in our study, the presence of these phenolic acids had no limiting effect on the production of biomass by *F. verticillioides* that tends to be higher in presence of the CE from pileus, but they may be involved in decreasing fumonisins and FA production per unit of biomass.

Sinapic acid have been shown to affect the production of trichothecenes by *F. culmorum* and *F. graminearum*, which also respond by stimulation of ergosterol. This effect might be indirect since treatment with sinapic acid led to dramatic accumulation of its biosynthetic precursor ferulic acid, and chlorogenic acid biosynthesis [59]. Both compounds have caffeic acid as a common precursor, and an increased accumulation of caffeic acid may occur in the early incubation period of fungi in the presence of sinapic acid [59]. On the other hand, *F. verticillioides* was shown to be able to biotransform chlorogenic acid into caffeic acid that is very efficient in inhibiting fumonisins biosynthesis [30]. The observed difference in sinapic acid composition between extracts from stipe and pileus of *L. edodes* and its correlation with the production of fumonisins might be associated with a transitory accumulation of caffeic acid having an inhibiting effect. However, no sinapic acid was detected in extracts of As-P that inhibited the accumulation of fumonisins. So, other phenolic compounds may contribute to the inhibition of fumonisins biosynthesis.

The significant difference in inhibiting activity of extracts from stipe and pileus of *L. edodes* could be also associated with an overall difference in composition observed with the profiles of ions detected by liquid chromatography coupled to accurate mass spectrometry. None of the compounds tentatively identified resulted as specific of stipe or pileus and they have not been reported in the literature for their effect, neither on the production of fumonisins and FA by *F. verticillioides* nor on the production of other mycotoxins. However, it is noteworthy that phenylalanine identified in CEs of pileus of *L. edodes* at a significantly higher level than in stipes is also a precursor of the synthesis of caffeic acid, through the conversion into *t*-cinnamic and *p*-coumaric acids [59]. Moreover, farnesyl acetone was identified only in stipe extracts. This compound is a sesquiterpenoid commonly found in plant essential oils and plant extracts, having antimicrobial and antioxidant activities [60,61]. In animal cells, it has been shown to block DNA replication [62], but there is no published data on a putative effect on the production of fumonisins or fusaric acid by fungi. The remaining metabolomic profiles deserve to be more deeply exploited to identify specific molecules to each CE that could be candidates for explaining their differences as inhibitors of the production of fumonisins and fusaric acid by *F. verticillioides*, and, when available, their activity could be tested in concentration response analyses.

## 4. Conclusions

The present results support the hypothesis that cultivable edible mushrooms have a potential to be a source of natural products for inhibiting the production of fumonisins and FA by *F. verticillioides.* Using methanolic extracts of stipes and pilei from the same sporophores of *L. edodes*, few molecules have been pointed as candidates for explaining differences in activity between methanolic extracts. In coming works, the use of other extracts and a deeper exploitation of metabolic profiles will reinforce our ability to identify efficient combinations of molecules in the extracts responsible for the inhibition or the stimulation of production of both mycotoxins. In addition, bioassay-guided isolation of active compounds in fractions of the active CEs will be accomplished in order to contribute to the identification of novel antifungals and to be able to produce natural mushroom extracts with high anti-mycotoxin activities. As the biological origin of a compound or a more or less purified extract does not fully guarantee its safety, it will be necessary to evaluate their toxicity and thereby ensure the safety of such products before offering them for use in greenhouse or field conditions. A limit to the future development of such extracts as bio-control products could be the production cost and competition with food uses of mushrooms. However, because of their degrading capacities, it is possible to cultivate these mushrooms intended for the production of extracts on contaminated substrates and thus recycle them [35].

## 5. Materials and Methods

### 5.1. Fungi

Strains of *Agaricus subrufescens* (IE-832), *Lentinula edodes* (IE-40), and *Pleurotus ostreatus* (IE-38) were provided by the Unidad de Biotecnología de Hongos Comestibles y Medicinales of the Instituto de Ecología A.C. (UBHCM, INECOL), Veracruz, Mexico.

Two *Fusarium verticillioides* strains were used in the experiments. The strain of *F. verticillioides* Fv63 had been isolated from maize cultivated in France. It was provided by Research Unit Mycology and Food safety (MycSA), INRAE, France. A specimen is deposited at the International Centre for Microbial Resources—Filamentous Fungi (CIRM-CF) collection (Marseille, France), under the reference number BRFM 2251. The second strain of *F. verticillioides* (MY3) had been isolated from maize cultivated in Mexico. The strain of MY3 belongs to the fungal collection at Dr. Javier Plasencia’s laboratory (Department of Biochemistry, Faculty of Chemistry, UNAM, Mexico City) and it was kindly provided by Dr. Diana Sánchez-Rangel (INECOL).

### 5.2. Cultivation of Mushrooms

The spawn for the three mushroom strains was prepared with sorghum grains and peat moss 2%. Both components were soaked with tap water for 24 h, and pre-cooked at 80 °C for 15 min. The peat moss and grains were drained for 1 h, mixed in bags, and sterilized for 1.5 h. Finally, the substrate was inoculated with plugs of mycelium from potato dextrose agar medium (PDA) and incubated at 25 °C in darkness until the substrate was completely invaded.

The mushrooms were cultivated with different techniques at UBHCM. The cultivation of *A. subrufescens* was performed as in Martos and co-workers [63]. The substrate used was a commercial compost prepared for commercial production of the button mushroom *A. bisporus*, and delivered by Altex Rioxal, Veracruz, México. Wheat straw and poultry manure were the main ingredients for composting (55% and 27%, respectively). Under aseptic conditions, 4 kg of compost were inoculated with 200 g of spawn and incubated at 25 °C in darkness. When the mycelium invaded the substrate completely (46 days after inoculation), the compost was covered with 5 cm of a casing layer containing sand 30%, peat moss 14%, limestone 35%, pulverized pumice 14%, and water 7%. After casing layer colonization, all crops were placed into a ventilated production room at 25 °C, and the relative humidity was always kept above 70%.

To obtain the *L. edodes*’ fruiting bodies, this fungus was cultivated following the technique recommended by Mata and Savoie [64]. Sixty grams of spawn were inoculated into 1.4 kg of barley straw added with 5% of oak sawdust and incubated at 25 °C in darkness conditions for about 50 days. Then, the crops were placed into a production room at 18 °C, 75–85% relative humidity, and with controlled ventilation.

Finally, *P. ostreatus* was cultivated according to Guzmán et al. [65]. Two thousand grams of spawn were inoculated into 4 kg of barley straw and incubated at 25 °C for 30 days. Subsequently, the crops were moved into the production room with the same conditions as for *L. edodes* cultivation.

Mushrooms were harvested at the commercial stage of development and immediately conditioned. Sporophores of *A. subrufescens* and *L. edodes* were separated by pileus and stipe, but not for *P. ostreatus*, due to the fact that both structures are not well differenced. They were frozen at −80 °C immediately after harvesting.

### 5.3. Production of CEs from Sporophores

Each part of the sporophore was freeze-dried (LABCONCO^®^ FreeZone 2.5, Kansas City, MO, USA) for 48 h and later ground to a powder (Osterizer^®^ 465-42, Mexico city, Mexico). Dried and pulverized samples (44.0 and 73.9 g for As-P and As-S respectively, 137.0 and 59.9 g for Le-P and Le-S respectively, and 80.0 g for Po) were macerated using MeOH (1:10 w/v) and shaking at 350 rpm on a magnetic stirrer (StableTemp™, Lakeside, CA, USA). The extractions were set in darkness at 21 °C and during 24 h. Afterwards, the extraction liquors were recovered by filtration and the solvent was eliminated in a rotary evaporator under reduced pressure (BÜCHI Rotavapor RII) at 40 °C to yield the corresponding CEs. The recovered mushroom materials were subjected to a second extraction procedure by using the same protocol and these CEs were combined with those obtained in the first extraction.

### 5.4. Cultivation of F. verticillioides in Presence of Muschroom CEs

*Fusarium verticillioides* Fv63 was cultivated on PDA in tubes and incubated at 25 °C in darkness for 7 days. Thereafter, 6 mL of sterile distilled water was added to each culture, stirred, and diluted to obtain suspensions that contained 3.3 × 10^7^ spores per mL. The My3 strain was incubated at 27 °C for 7 days, and a suspension of 1 × 10^6^ spores per mL was prepared.

A glucose, amylopectin, yeast extract, and peptone (GAYEP) medium was obtained by mixing 900 mL of a solution containing 40 g L^−1^ glucose and 10 g L^−1^ amylopectin from maize (autoclaved for 30 min at 105 °C) and 100 mL of a solution containing 1 g L^−1^ neopeptone and 1 g L^−1^ yeast extract, autoclaved for 20 min at 121 °C [54]. It was used for the mycotoxin production by the *F. verticillioides* strains.

For each strain, 100 μL of spore suspensions were added in 25 mL flasks containing 10 mL of GAYEP medium [54]. The extracts (As-P, As-S, Le-P, Le-S, and Po) were dissolved in water, which was used as the vehicle, and independently added to the medium at a concentration of 2 mg mL^−1^. The spore suspension was inoculated into GAYEP with water as control. Fv63 was incubated with shaking (150 rpm) at 25 °C for 14 days and My3 was incubated with 125 rpm at 27 °C for 14 days. For each experiment, cultures were made in triplicate.

### 5.5. Mycelial Biomass Quantification

For separating biomass, each culture was decanted into previously weighed 15 mL tubes. Then, they were centrifuged at 5000 rpm and 4 °C for 10 min. The fungal biomass was frozen at −80 °C and the supernatant was stored at −20 °C until mycotoxins were quantified. Fungal biomass was freeze-dried for 48 h and weighted.

### 5.6. Analyses of Mycotoxins

For the quantification of FB1, FB2, and FB3, fumonisins were extracted from culture media (supernatants) as described previously by Picot et al. [54]. Firstly, the pH of each sample was adjusted between 5.8 and 6.5 using sodium hydroxide (Sigma-Aldrich, Toluca, Mexico) (1 N) and hydrochloric acid (Sigma-Aldrich) (1 N) solutions. An ion exchange column (Blond Elut LRC-SAX 500 mg Agilent technologies) was washed with 5 mL of MeOH high-performance liquid chromatography (HPLC)-grade (Interchim, Montluçon, France), and then 5 mL MeOH/Milli Q water (3:1, v:v). After column conditioning, 5 mL sample were added, and rinsed successively with 5 mL MeOH/Milli Q water (3:1, v:v) and 3 mL MeOH. Finally, fumonisins were eluted with 10 mL MeOH/acetic acid 1%. The solvent evaporation was achieved in a heating system at 60 °C under nitrogen flow. Sample extracts were resuspended in 200 μL of MeOH HPLC-grade, stirred for 30 s, and filtered through a 0.2 μm filter. Fumonisin concentrations were determined using a Shimadzu Prominence ultra-high-performance liquid chromatography system (UHPLC) chain composed of two LC-30AD pumps, and one autosampler (SIL-30AC) coupled to a fluorescence detector (RF-20A), where the derivatization with o-phthalaldehyde (OPA) and β-mercaptoethanol was performed automatically to make the fumonisins fluorescent. The mobile phase was 77% of MeOH in HPLC-grade with 23% of NaH_2_PO_4_ 2H_2_O 0.1 mol L^−1^. The flow was 1.2 mL min^−1^. The injection volume was 5 μL. The column was a Phenomenex, Kinetex C18, 150 × 4.5 mm, 2.5 μm. Retention times of detected toxins were compared with commercial standards of FB1, FB2, and FB3 (Romer Labs, Austria). A calibration curve for each fumonisin was made in a concentration range of 1 to 50 ppm (R^2^ values ≥ 0.97 were considered for the linearity range). Data were expressed as μg of excreted fumonisin per g of dry biomass.

FA and FB1 were analyzed with a 1290 infinity Agilent UHPLC coupled to a 6460 Agilent triple quadrupole (QqQ) mass spectrometer (MS) with a dynamic multiple reaction monitoring (dMRM) method. The transition for FA and FB1 were 722.4 > 352.3 and 180.1 > 134.1 respectively, with collision energy of 20 and 40 V, respectively. The polarity used for both compounds was positive and the ionization source was electrospray. Tubes containing 5 mL of culture medium (supernatant) were freeze-dried for 48 h. Each obtained extract was dissolved in 1 mL of MeOH ≥99.9% LC-MS ultra CHROMASOL^®^ for UPLC (Sigma-Aldrich) with 0.1% of formic acid LC-MS grade (Sigma-Aldrich) by stirring for 5 min, sonicated for 5 min, and stirred for 30 s more. All samples were filtered through a 0.2 μm filter. For UHPLC separation, the mobile phases were water with 0.1% of formic acid (A solution) (MS-grade, SIGMA-ALDRICH) and a solution of acetonitrile 90% with 0.1% of formic acid (MS-grade, SIGMA-ALDRICH) (B solution) (Table 3). The flow was 0.3 mL/min. The injection volume was 1 μL. The column was an Agilent, Zorbax SB-C18, 2.1 × 50 mm, 1.8 Microns. The column temperature was 40 ± 0.8 °C.

The gradient elution was as follows: 1% B for 3 min, 1% to 99% B for 9 min, 99% B for 2 min, 1–99% B for 1 min. Mass spectrometry conditions were set as follow: the gas temperature was 325 °C and its flow rate was 6 L/min. Nebulizer was 45 psi. 350 °C of sheath gas temperature and its flow rate was 11 L/min. 3500 of capillary voltage and 500 of nozzle voltage.

Retention times of detected toxins were compared with standards of FB1 and FA (Sigma-Aldrich^®^). A calibration curve for each mycotoxin was made in a concentration range of 0.5 to 17 μM (R^2^ values ≥ 0.97 were considered for the linearity range). Data were expressed as μg of excreted FB1/FA per mg of dry biomass, and the percentage of inhibition of each treatment was calculated according to their respective negative control with the following equation:

$$\%\;\mathrm{inhibition}=100-\frac{\left(T\times 100\right)}{C}$$

where: T = mycotoxin concentration in the treatment, and C = mycotoxin concentration in the control.

### 5.7. Metabolomic Analysis and Chemical Profiling of the CEs

Solutions of 20 mg mL^−1^ of the six CEs were prepared in MeOH ≥ 99.9% LC-MS ultra CHROMASOL^®^ for UHPLC-MS (Sigma-Aldrich) 0.1% formic acid LC-MS-grade (Sigma-Aldrich). The samples were filtered with nylon membrane filters, with 0.2 μm pore size (Acrodisc^®^, Mexico City, Mexico). Finally, all samples were injected in a UHPLC (Acquity Class I, Waters™, Burnsville, MN, USA) coupled to a quadrupole time of flight (QTOF) high-resolution mass spectrometry analyzer (HRMS, Synapt G2-Si, Waters™).

The chromatography was performed on an Acquity BEH column (1.7 μm, 2.1 × 50) with a column oven and sample temperatures of 40 and 15 °C, respectively. The mobile phase consisted of water and acetonitrile, both with 0.1% formic acid (Sigma). The gradient conditions of the mobile phases were 0–13 min liner gradient 1–80% of acetonitrile/0.1% formic acid (total run time: 20 min). The flow rate was 0.3 mL min^−1^ and 1 μL of extract was injected. The mass spectrometric analysis was achieved with an electrospray ionization (ESI) source in positive mode with a capillary, sampling cone, and source offset voltages of 3000, 40, and 80 V, respectively. The source temperature was 100 °C and the desolvation temperature was 20 °C. The desolvation gas flow was 600 L/h and the nebulizer pressure was 6.5 Bar. The conditions used for MS analysis were mass range 50–1200 Da, function 1 CE, 6 V, function 2 CER 10–30 V, and scan time 0.5 s.

For tentative identification of the metabolites from CEs with contrasted biological activity, a search of chemical compounds being present either in stipes or pilei of *L. edodes*, but not in both, was made using MassLynx software (Waters^®^) by the comparison of the *m*/*z* values of the precursor and products ions with those reported in the spectral databases, such as METLIN and FooDB (metlin.scripps.edu and foodb.ca, respectively). A mass accuracy error lower than 5 ppm was accepted.

### 5.8. Analysis of Phenolic Compounds

A total of 60 specific phenolic compounds were searched for identification and quantification in all CEs from sporophores by using the protocol reported by Juárez-Trujillo et al. [66]. All samples were analyzed by UHPLC-MS-QqQ with a dMRM as an acquisition method [66].

The chromatographic analysis was carried out on a ZORBAX SB-C18 column (1.8 μm, 2.1 × 50 mm; Agilent Technologies) with the column oven temperature at 40 °C. The mobile phase consisted of (A) water and (B) acetonitrile, both containing 0.1% formic acid. The gradient conditions of the mobile phase were: 0 min 1% B, 0.1–40 min linear gradient 1–40% B, 40.1–42 min linear gradient 40–90% B, 42.1–44 min isocratic 90% B, 44.1–46 min linear gradient 1–90% B, 46.1–47 min 1% B isocratic (total run time 47 min). The flow rate was 0.1 mL/min, and 5 μL was the sample injection volume. The ESI source was operated in positive and negative ionization modes. The desolvation temperature was 300 °C, the cone gas (N2) flow was 5 L/min, the nebulizer pressure was 45 Psi, the sheath gas temperature was 250 °C, the sheath gas flow was 11 L/min, the capillary voltage (positive and negative) was 3500 V, and the nozzle voltage (positive and negative) was 500 V. The fragmentor voltage was 100 V and the cell accelerator voltage was 7 V for all compounds. The identity was corroborated by co-elution with authentic standards under the same analytical conditions above described for each phenolic compound. A calibration curve in a concentration range of 0.5–17 μM was prepared for quantitation of each compound (R^2^ values ≥ 0.97 were considered for the linearity range). The dMRM transition for each compound was reported by Juárez-Trujillo et al. [62]. The data were processed using the MassHunter Workstation software, version B.06.00 (Agilent Technologies, Santa Clara, CA, USA), and the results were expressed as μg g^−1^ of CE.

### 5.9. Data Analyses and Statisitics

A factorial design housed in a completely randomized design were accomplished in the experimental stage. In order to determine some differences among CEs with respect to the control, the one-way analysis of variance (ANOVA) was used with R Studio^®^ software (version 3.6.1, RStudio, Boston, MA, USA), at a level of significance set at 0.05. Means for groups in homogeneous subsets were determined using the Tukey’s multiple comparisons test (Tukey’s post hoc test), at the 95% confidence interval. Transformation data (log10) was carried out when the results did not show normality (Shapiro–Wilk’s test) or homoscedasticity (Bartlett’s test) among treatments. All data were presented as mean values with their standard deviation indicated (mean ± SD).

In the untargeted metabolomics approach, differences between the metabolomes of the five CEs were visualized with a PCA using the retention times and the *m*/*z* values obtained from the UHPLC-HRMS-QTOF analysis. After PCA, a volcano plot analysis was achieved to compare the datasets of the two CEs from *L. edodes* (stipe and pileus) and point out which metabolites were significantly different between the samples. The volcano plot also calculated fold change and *p*-values to allow a comparison of absolute value changes between the two group of means. The variable was reported as significant if the value was above a fold change of 2, and the level of significance was set at 0.05. Both analyses were carried out with four replicates. The PCA was accomplished with the MassLynx and MarkerLynx softwares (Waters™) and the volcano plot with the Metaboanalyst 4.0 software (metaboanalyst.ca, Montreal, Canada). All variables were Pareto-scaled prior to analyses.

## Figures and Tables

**Table 1:** Dry biomasses of mushroom, crude extracts obtained (CE), and extraction yields obtained from *A. subrufescens* pileus (As-P) and stipe (As-S), *L. edodes* pileus (Le-P) and stipe (Le-S), and *P. ostreatus* sporophore (Po).

Strain	Sporophore Structure	Dry Biomass (g)	CE (g)	Yield (%)
IE-832	As-P	44.0	10.1	23.0
	As-S	73.9	9.8	13.2
IE-40	Le-P	137.0	25.2	18.4
	Le-S	59.9	17.1	28.5
IE-38	Po	80.0	23.7	29.6

**Table 2:** Metabolites putatively identified in the CEs from *L. edodes* that are significantly present in only one part of the sporophore. Levels of metabolite identification (LMI) [36] is included as well as mass-to-charge ratio (*m*/*z*), the adducts, and retention time (RT) of each compound.

Metabolite	LMI	Formula	*m*/*z* Experimental	*m*/*z* Theoretical	Error (ppm)	Adduct	RT	Product Ions (*m*/*z*)	CE
L-Phenylalanine	2	C_9_H_11_NO_2_	166.0864	166.0863	−0.6	[M + H]^+^	1.66	120.0807 103.0536	Pileus
Adenine	2	C_5_H_5_N_5_	136.0618	136.0618	0.0	[M + H]^+^	1.94	119.0349 92.0234	Pileus
3-amino-2-naphthoic acid	2	C_11_H_9_NO_2_	188.0712	188.0706	−3.2	[M + H]^+^	2.30	170.0601 142.0651	Pileus
(22E,24x)-Ergosta-4,6,8,22-tetraen-3-one	3	C_28_H_40_O	393.3142	393.3152	2.5	[M + H]^+^	18.46	No *	Pileus
Farnesyl acetone	2	C_18_H_30_O	245.2272	245.2269	1.2	[M + H-H_2_O]^+^	16.39	107.0847 109.1005 123.1160	Stipe

*: no ions were found for this metabolite.

**Table 3:** Phenolic compounds identified and quantified in CEs from *A. subrufescens*, *L. edodes* and *P. ostreatus* sporophores.

Species and Sporophore Structure	Phenolic Compounds (μg g^−1^ of CE)						
	ShA	CiA	PrA	HyBA	SiA	HyPA	Ch
As-P	44.6 ± 2.4	N/D	7.9 ± 0.1	6.2 ± 0.6	N/D	N/D	131.1 ± 0.4
As-S	N/D	N/D	7.4 ± 0.1	7.9 ± 0.2	8.3 ± 0.3	N/D	135.0 ± 0.7
Le-P	34.5 ± 9.4	N/D	6.2 ± 4.9	37.0 ± 0.6	7.7 ± 0.7	9.2 ± 1.1	130.4 ± 0.3
Le-S	24.4 ± 1.5	N/D	6.2 ± 4.4	11.3 ± 0.2	N/D	5.2 ± 0.4	130.7 ± 0.4
S/P	71%		100%	30%	0%	56%	100%
Po	42.3 ± 2.6	5.0 ± 0.2	7.9 ± 0.1	7.7 ± 0.3	5.0	5.6 ± 0.6	130.4 ± 1.1

Phenolic compounds: shikimic acid (ShA), *trans*-cinnamic acid (CiA), protocatechuic acid (PrA), 4-hydroxy-benzoic acid (HyBA), sinapic acid (SiA), 4-hydroxy-phenylacetic acid (HyPA), and chrysophanol (Ch). Not detected (N/D).
